# Gelatinous Transformation of Bone Marrow Following Lymphoma and a Novel, Potential Treatment

**DOI:** 10.7759/cureus.59354

**Published:** 2024-04-30

**Authors:** Logan A Hahn, Emina Torlakovic, Mark Bosch

**Affiliations:** 1 Internal Medicine, University of Saskatchewan, Saskatoon, CAN; 2 Hematopathology, University of Saskatchewan, Saskatoon, CAN; 3 Hematology, Saskatchewan Cancer Agency, Saskatoon, CAN

**Keywords:** bone marrow failure, lymphoma, stem cell transplant, pancytopenia, gelatinous transformation of bone marrow

## Abstract

Gelatinous transformation of bone marrow (GTBM) is a rare hematologic condition in which hematopoietic cells in the bone marrow are replaced by extracellular gelatinous substances, often resulting in cytopenias. The true incidence of this condition is presently unknown, as the current body of literature primarily consists of case reports. However, an analysis of a large bone marrow registry suggests that this is a highly rare entity even among a population requiring bone marrow biopsy. We present a case of a 24-year-old man with a history of diffuse large B cell lymphoma and an associated 45-kilogram weight loss, who was later found to have GTBM. The extent of his cytopenias resulted in a prolonged hospitalization with numerous complications, eventually leading to experimental treatment with allogeneic stem cell transplantation (ASCT). To our knowledge, this is the first reported case of GTBM in which ASCT was employed as a potential treatment modality. While our patient did have clinical improvement following ASCT, the permanence of these results is presently unclear. Furthermore, it is uncertain if the ASCT was truly causative of the stabilization of the patient. Given this, we are currently unable to advocate for ASCT as a treatment for GTBM. We report this case to raise awareness of this rare entity in the context of refractory cytopenias.

## Introduction

Gelatinous transformation of bone marrow (GTBM) is an exceptionally rare condition whereby hematopoietic cells in the bone marrow are replaced by extracellular gelatinous substances similar to mucopolysaccharides [1]. As such, the main manifestation of this disease is often severe cytopenias. The condition was first described in patients with anorexia nervosa in 1930 but has now been documented in a multitude of disease states including celiac disease, congestive heart failure, and acquired immunodeficiency syndrome (AIDS) [2,3]. The true incidence of the disease is presently unknown; however, retrospective analysis of a large bone marrow registry (n=80,000) only documented the finding in 0.2% of the analyzed specimens [3]. Case reports of the condition have rapidly increased in the past few decades, perhaps indicating a growing awareness of the disease [2]. Today, malignancy and malnutrition are recognized as the most common etiologies of GTBM [3]. While GTBM was initially described in cases of solid tumor cancers, case reports have now documented the pathology in various hematologic conditions such as multiple myeloma, acute myeloid leukemia, acute lymphocytic leukemia, chronic myeloid leukemia, and myelofibrosis [4-9].

Mortality and treatment approaches are ill-defined with respect to GTBM; however, it appears that the pathologic bone marrow changes are reversible in some patients. Case reports, for instance, have documented the resolution of GTBM following treatment of the underlying disease, balanced nutrition, and support with hematopoietic growth factors [10-12]. We hereby present a case of a 24-year-old man with a history of diffuse large B cell lymphoma (DLBCL) who was found to have GTBM, which we attempted to treat with allogeneic stem cell transplantation (ASCT).

This article was previously posted to the ResearchSquare preprint server on September 14, 2023.

## Case presentation

A 24-year-old male from rural Saskatchewan presented to care at the Royal University Hospital in Saskatoon, Saskatchewan, Canada, after a yearlong history of fever, chills, and a 45-kilogram weight loss. His comorbidities were notable for type 1 diabetes and psoriasis. His initial investigations in December 2021 were significant for profound bicytopenia and neutropenia (Table 1). A pan-computed tomography (CT) scan demonstrated widespread lymphadenopathy through the neck and mediastinum, as well as the retroperitoneum and periportal regions. A bone marrow biopsy and aspirate from the left iliac crest and a core lymph node biopsy from an inguinal lymph node were subsequently arranged, which revealed DLBCL and markedly decreased trilineage hematopoiesis. Fluorescence in situ hybridization was negative for MYC, BCL-2, and BCL-6.

**Table 1:** Complete blood count and differential at admission

Complete Blood Count Parameter	Value	Reference Range (Units)
Hemoglobin	55	135-180 (g/L)
Mean cell volume	78	79-97 (fL)
Platelets	10	150-400 (x10^9^/L)
Leukocytes (total)	4.5	4-11 (x10^9^/L)
Neutrophils	0.4	1.5-7.5 (x10^9^/L)
Lymphocytes	4.1	1.1-4.4 (x10^9^/L)
Eosinophils	0.0	0.0-0.6 (x10^9^/L)
Basophils	0.0	0.0-0.2 (x10^9^/L)
Monocytes	0.0	0.2-0.8 (x10^9^/L)

Due to the patient’s ongoing transfusion requirements, he was admitted to the oncology ward to begin chemotherapy. He received his first cycle of rituximab plus cyclophosphamide, doxorubicin, vincristine, and prednisone (R-CHOP) at the end of December 2021. The patient completed his sixth course of R-CHOP chemotherapy in July 2022 with a follow-up positron-emitted tomography (PET) scan revealing a complete response to chemotherapy (Deauville stage 3). Despite a successful treatment regimen, the patient had multiple requirements for ongoing hospital admission.

Firstly, the patient had refractory cytopenias requiring almost daily support with transfusion products and hematopoietic agents such as granulocyte colony stimulating factor (G-CSF). From December 2021 to September 2022, the patient required more than 140 units of packed red blood cells and 100 units of adult-dose platelets. His persistent neutropenia unfortunately precipitated several nosocomial infections, which further complicated his course. He was treated for prolonged febrile neutropenia for which a dental abscess was ultimately believed to be the culprit source, hepatosplenic candidiasis, COVID pneumonia, and vancomycin-resistant enterococcal line infection.

**Figure 1:**
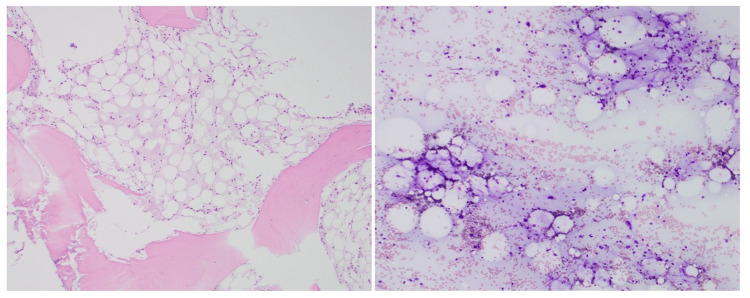
Gelatinous transformation of bone marrow A bone marrow biopsy of the patient performed in February 2022 demonstrating gelatinous transformation of bone marrow. The bone marrow is markedly hypocellular with no definite evidence of hematopoeisis. Gelatinous transformation was seen universally in all bone marrow fragments.

A possible explanation for the patient’s cytopenias came into view, with repeat bone marrow biopsies in February and August 2022 demonstrating persistent GTBM (Figure 1). While the expected course of GTBM has not been well-explored, the recrudescence of the patient’s disease was thought to be peculiar as the patient’s lymphoma and associated malnutrition had been treated. Given the patient’s young age, the consequences of his cytopenias, and separation from his home community, a strong impetus to ameliorate his refractory GTBM developed.

In September 2022, the patient was approached for ASCT as a potential treatment for his GTBM. To our knowledge, this modality has never been used exclusively for the treatment of GTBM. He underwent a conditioning regimen and underwent transplantation later in the month (Table 2). The patient tolerated the transplantation well; however, he had persistent neutropenia. Eventually, he experienced some recovery following support with G-CSF. His chimerism showed less than 10% donor cells, indicating transplant failure. Nonetheless, the patient experienced enough recovery in his cell counts to be safely discharged from the hospital in November, after nearly a year of hospitalization.

**Table 2:** Allogeneic transplant characteristics FluCyATG, fludarabine, cyclophosphamide, and antithymocyte globulin; CMV, cytomegalovirus; EBV, Epstein-Barr virus; VZV, varicella-zoster virus; HSV, herpes simplex virus; GVHD, graft versus host disease; MMF, mycophenolate mofetil; ATG, antithymocyte globulin

Conditioning Regimen	FluCyATG
Donor type	Matched unrelated donor (12/12)
ABO (donor/recipient)	O positive/O positive
CMV (donor/recipient)	Positive/negative
EBV (donor/recipient)	Positive/negative
VZV recipient	Positive
HSV recipient	Positive
Initial chimerism	<10%
GVHD prophylaxis	Tacrolimus, MMF, ATG

As of the time of this writing, this patient has not required re-admission to the hospital and is no longer transfusion-dependent. However, he has required three donor lymphocyte infusions to assist with engraftment. Given his ongoing leukopenia, he receives no prophylactic immunosuppression to prevent graft versus host disease (GVHD). Fortunately, he has not had any symptoms consistent with GVHD at this point. His most recent PET scan in April 2023 showed continued remission of his lymphoma. He has close follow-up arranged via our institution’s cancer center and has returned to his home community, which is far away from a tertiary center in Saskatchewan.

## Discussion

There is a great deal of uncertainty surrounding this unusual case of refractory GTBM. Firstly, it is unclear as to the exact etiology of GTBM in this patient. Major hypotheses include lymphoma, weight loss, or perhaps R-CHOP chemotherapy itself.

In the small body of literature surrounding GTBM, malnutrition and starvation are frequently reported as the underlying etiology [2,3,11-15]. It has been theorized that starvation-associated catabolism may lead to marrow fat mobilization and replacement with mucopolysaccharides [16]. A study conducted in a cohort of patients with anorexia and GTBM even demonstrated a correlation between the histologic burden of disease and the degree of weight loss [13]. Perhaps this indicates that cachexia, malnutrition, and malabsorption underlie the pathogenesis of GTBM. Numerous case reports have documented resolution of GTBM with nutritional support and weight gain [10,12,14,15]. Given these strong associations in the literature between nutritional status and GTBM, it is somewhat peculiar that our patient did not have initial hematologic recovery despite improvement in his nutritional status as an inpatient.

Perhaps hematologic malignancy plays an independent role in the pathogenesis of GTBM by somehow altering the bone marrow microenvironment. Case reports of GTBM associated with hematologic malignancy have been steadily climbing in recent decades [2,4,7-9]. Certainly, many patients with hematologic malignancy also have profound malnutrition and malabsorption. However, there are case reports of GTBM occurring in acute and chronic leukemia patients without apparent cachexia or malnutrition [8,17]. Again, we would have expected our patient to have some hematologic recovery after attaining a complete response to R-CHOP.

Chemotherapeutic regimens have also been speculated to play some role in the pathogenesis of GTBM. For instance, imatinib and melphalan have been implicated in the development of GTBM in case reports [4,8,18]. To our knowledge, there is only one other case report of a patient developing GTBM following R-CHOP therapy [19]. However, the patient in this case report presented with a prodrome of anorexia, which may confound the association. A mechanistic role for chemotherapy-induced GTBM has not been substantiated.

Lastly, we are uncertain if resolution of GTBM has truly occurred in our patient post-ASCT. Certainly, hematopoiesis has improved from when the patient was admitted at our institution and required near daily transfusion support. However, he remains pancytopenic at the time of this writing. Nonetheless, there are reports that the severity of cytopenias has poor correlation with the histologic burden of disease [13]. At the time of this writing, we have not subjected the patient to repeat bone marrow biopsy, as this would be unlikely to alter clinical management given the fact that there are currently no specific treatments described for GTBM.

## Conclusions

Case reports of GTBM are steadily increasing and the pathologic bone marrow state is now considered to be a rare consequence of a multitude of disease states, thereby indicating that this condition has been previously under-recognized. Further exploration of the pathogenesis and epidemiology of GTBM is likely needed to unveil new treatment options for this rare disease. At this moment, we recommend that clinicians give some consideration to this diagnosis and strive for aggressive amelioration of the underlying cause. While ASCT may have been the factor that stabilized our patient and enabled him to be weaned off transfusion support, a much deeper understanding of the disease process is required before we can advocate for this approach to be widely used in the context of refractory GTBM.
